# MEMS-Casting Fabricated Chip-Style 3D Metal Solenoidal Transformers towards Integrated Power Supply

**DOI:** 10.3390/mi13020325

**Published:** 2022-02-18

**Authors:** Nianying Wang, Changnan Chen, Pu Chen, Jiebin Gu, Pichao Pan, Ruofeng Han, Min Liu, Xinxin Li

**Affiliations:** 1 State Key Laboratory of Transducer Technology, Shanghai Institute of Microsystem and Information Technology, Chinese Academy of Sciences, Shanghai 200050, China; wangny@mail.sim.ac.cn (N.W.); ccn@mail.sim.ac.cn (C.C.); j.gu@mail.sim.ac.cn (J.G.); panpichao@mail.sim.ac.cn (P.P.); hanruofeng@mail.sim.ac.cn (R.H.); liumin@mail.sim.ac.cn (M.L.); 2 School of Information Science and Technology, ShanghaiTech University, Shanghai 201210, China; 3 School of Microelectronics, University of Chinese Academy of Sciences, Beijing 100049, China; 4 East China Institute of Photo-Electron IC, Bengbu 233030, China; chenpu520033@163.com

**Keywords:** 3D metal solenoid, AC-DC transformer, micro-casting, on-chip integrated power supply, LED lighting

## Abstract

A silicon-chip-based 3D metal solenoidal transformer is proposed and developed to achieve AC-DC conversion for integrated power supply applications. With wafer-level micro electromechanical systems (MEMS) fabrication technique to form the metal casting mold and the following micro-casting technique to rapidly (within 6 min) fill molten ZnAl alloy into the pre-micromachined silicon mold, 45-turns primary solenoid and 7-turns secondary solenoid are fabricated in silicon wafers, where the two intertwining solenoids are located at inner deck and outer deck, respectively. Permalloy soft magnetic core is inserted into a pre-etched channel in the silicon chip, which is surrounded by the solenoids. The size of the chip-style transformer is as small as 8.5 mm × 6.6 mm × 2.5 mm. The internal resistance of the primary solenoid is 1.82 Ω and that of the secondary solenoid is 0.16 Ω. The working frequency of the transformer is 60 kHz. Combined with the testing circuit of the switch mode power supply, the DC voltage of 13.02 V is obtained when the input is 110 V at 50 Hz/60 Hz. Furthermore, the on-chip 3D solenoidal transformer is used for lighting four LEDs, which shows great potential for AC-DC power supply. The wafer-level fabricated chip-style solenoidal AC-DC transformer for integrated power supply is advantageous in uniform fabrication, small size and volume applications.

## 1. Introduction

The power supplies are widely used in various electronic applications, such as Light Emitting Diodes (LEDs) [1,2], sensors [3], wearable electronics [4,5], medical devices [6], smart phones [7] and so on. Miniaturization of the power supply has become the main focus for developing future generation integrated power supplies. The transformer in power supply is the major contributor to the volume and weight of the power supply. Great efforts have been made to reduce the size of the power supply, such as commercial switch mode power supplies (SMPS), which are one of the most successful applications to greatly reduce the size of the transformer. According to the voltage conversion mechanism, the converters for power supplies can be classified as DC to DC [8] and AC to DC converters [9,10,11]. Nowadays, almost all the electronic devices are powered by conventional AC-based lighting grids such as AC 110 V or 220 V at 60 Hz or 50 Hz. The AC-based electricity should be transformed into a DC source by power supply before it can be used for powering the electronic devices.

In order to study the power supply for electronic devices, researchers have made great efforts in transformer design [12,13,14,15] and circuit design including the circuit topology towards flyback converter and forward converter [16,17,18,19,20,21,22,23,24,25,26]. As one of the most important modules of DC-DC or AC-DC conversion, almost all the commercial transformers are fabricated by metal wire winding that suffers large size and non-batch fabrication. This hinders miniaturization and chip-based integration of transformers.

This paper proposes 3D metal solenoidal transformers that are fabricated with silicon-wafer-based integration method. First, silicon mold wafers for the 3D metal solenoid are fabricated by MEMS techniques including through-wafer deep reactive ion etch (DRIE). Then, the pre-fabricated silicon mold, which consists of six-layer silicon wafers, is aligned and stacked together for molten alloy filling (i.e., micro-casting). With the wafer-level micro-casting technique [27] that were ever used for forming through-silicon-vias (TSVs), the molten ZnAl alloy (with the melting point as low as 380 °C) is filled into the molds of the whole wafer within 6 min. After cooling, demolding and saw-dicing, the chip-based 3D metal solenoid/channel structures are formed. By assembling two E-shaped soft magnets into the hollowed channel inside the core of the solenoid chip, which was previously formed during the micromachining process for the silicon mold. In the following sections, we will describe design, fabrication and testing details of the proposed on-chip integrated transformer. In future work, if the other semiconductor devices/components for electric power circuit could be further integrated into the silicon chip that already contains the transformer, a chip-style integrated power-supply module would be realized. Now, the integration of the transformer coil into a silicon chip is an important step towards the integrated AC-DC power source.

## 2. Design and Modeling

**Figure 1:**
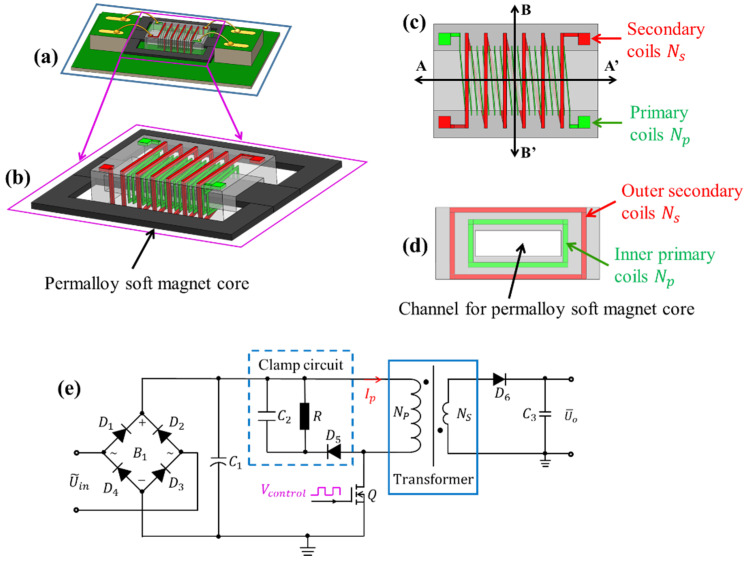
(**a**) Schematic prototype of the transformer for testing; (**b**) 3D structure of the chip-style transformer; (**c**) Top view of the transformer; (**d**) Cross view of the transformer along B-B’ direction defined in (**c**); (**e**) Circuit diagram of a switch mode power-supply module.

The prototype of the chip-based integrated transformer for power-source experiment is schematically shown in Figure 1a, which mainly consists of the 3D metal solenoidal transformer and the soft magnetic core. The inner-layer primary solenoid is surrounded by the outer-layer secondary solenoid (shown in Figure 1b). Two E-shaped specially fabricated commercial permalloy soft magnetic cores (Iron Nickel Alloy, 1J85) are directly inserted from the double sides into the central channel of the solenoid to increase the inductance of the solenoidal coils, where the initial permeability of the chosen permalloy is more than 30 mH/m and its maximum permeability can be up to 115 mH/m. The silicon mold for metal solenoid micro-casting and the central channel to accommodate the soft magnetic core are formed by stacking six micromachined silicon wafers and the 3D solenoid-coil intertwines inside the six silicon layers. The primary solenoidal coils (see the green-colored inner-layer solenoid in Figure 1d) and the secondary solenoidal coils (the red-colored outer-layer coil in Figure 1d) are clearly shown.

Figure 1e shows the circuit diagram of the power supply module. For the power supply testing experiment, the fabricated on-chip transformer will be connected to the circuit by replacing the traditional wire-wound transformer. AC input voltage ${\tilde{U}}_{in}$ is first transformed to DC voltage by the rectifier bridge $B_{1}$ and then input to the primary solenoid of the transformer. Controlled by oscillation of the switch transistor *Q*, the AC-to-DC conversion can be achieved.

**Figure 2:**
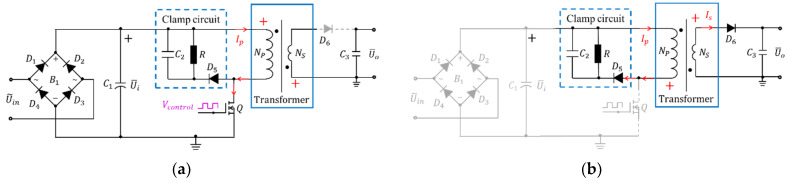
Two operating modes of the transformer for power supply. (**a**) When Q is switched ON. (**b**) When Q is OFF.

There are two operating modes since the circuit has only one switch. Shown in Figure 2a, the transformer acts as a forward transformer when the transistor *Q* is switched ON. The input supply charges the primary solenoidal coils, and no current flows in the secondary side since the diode $D_{6}$ is reversely biased. As shown in Figure 2b, the transformer acts as single-ended flyback transformer when the switch Q is OFF. The supply gets disconnected from the circuit and the energy stored in the primary solenoid is transferred to the secondary solenoid. At this time, the diode $D_{6}$ is forward biased and the voltage is stored in the output capacitor. Table 1 shows the symbols listed in Figure 2.

When Q is ON, the self-induced voltage by the primary solenoid can be expressed as

(1) $$e_{1}=L_{p}\frac{di_{p}}{dt}={\bar{U}}_{i}$$

where $e_{1}$ and $L_{p}$ represent self-induced voltage and inductance of primary solenoid. $i_{p}$ denotes the current flowing through the primary solenoid that can be expressed as

(2) $$i_{p}=\frac{{\bar{U}}_{i}}{L_{p}}+i_{p}\left(0\right)$$

When Q is switched to OFF, the current $i_{p}$ changes to zero suddenly. However, the magnetic flux of the magnetic core cannot immediately change to zero. The change of the magnetic flux will be constrained by the current flowing through the primary and secondary coils of the transformer. Therefore, when the switch Q is turned off, the magnetic flux in the transformer core is mainly determined by the current in the secondary solenoid, which can be expressed as

(3) $$e_{2}=-L_{s}\frac{di_{s}}{dt}={\bar{U}}_{o}$$

(4) $$i_{s}=-\frac{{\bar{U}}_{o}}{L_{s}}+i_{s}\left(0\right)$$

where $e_{2}$ represents self-induced voltage of secondary solenoid, $L_{s}$ is inductance of secondary solenoid, $i_{s}$ denotes current flowing through secondary solenoid.

**Figure 3:**
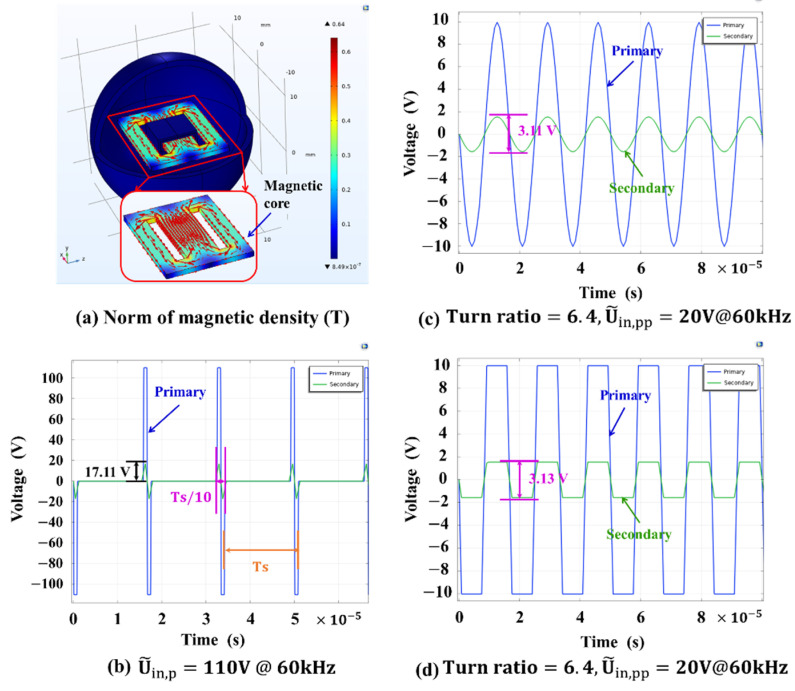
Dynamic response results obtained by using COMSOL simulation. (**a**) Norm of magnetic density in permalloy soft magnetic core when input voltage is applied to the primary solenoid; (**b**) The induced peak voltage of secondary solenoid is 17.11 V when 110 V AC voltage is applied to the primary solenoid; (**c**) Voltage transforming simulation result to confirm the voltage ratio of 6.4 is consistent with the designed turns ratio of 6.4 when sinusoidal voltage signal is applied to the primary solenoid; (**d**) Voltage transforming simulation result to confirm the voltage ratio of 6.4 is consistent with the designed turns ratio of 6.4 when rectangular voltage signal is applied to the primary solenoid.

Based on the designed parameters of the transformer shown in Table 2, the COMSOL simulation results are shown in Figure 3. For the COMSOL simulation, “Ampère’s Law” and “Coil” in “Magnetic Field” interface are used to set conditions for the transformer model, “Normal” mesh size is chosen for meshing and “Coil Geometry Analysis” for step 1 and “Time Dependent” for step 2 are used for the dynamic response simulation. Figure 3a shows that the flux linkage generated by the primary solenoid mainly flows through the high-permeability soft magnetic core due to the advantage of the solenoid structure. The simulated voltage ratio (${\tilde{U}}_{in}/{\tilde{U}}_{o}$) is consistent with the designed turns ratio shown in Figure 3c,d. Shown in Figure 3b, when the 110 V input AC voltage is under 60 kHz switching frequency and 0.1*T_s_ ($T_{s}=1/f_{s}$) duty cycle, the induced peak AC voltage of 17.11 V can be obtained at the secondary solenoid. The parameters in Table 2 are determined after repeated simulation for optimization.

## 3. Fabrication

Recently, our group developed a MEMS fabrication technique, named micro-casting, to fill molten metal into a pre-molded silicon wafer to form 3D metal solenoid structures that contain many turns of dense coils [28]. After filling the silicon mold with a low melting-point metal, the molten metal outside the solenoid structure can be pinched off at specifically designed filling nozzles, where the micromachined nozzles in a silicon nozzle wafer were designed into a slim shape with a high aspect ratio [28]. After filling, the solenoid wafer is cooled down and demolded from a top-cover wafer and the bottom nozzle wafer. In this way, lots of solenoid wafers can be sequentially fabricated, and the nozzle wafer and the top-cover can be repeatedly used. The technical details of the micro-casting equipment, the mold wafer preparation by using deep reactive ion etching (DRIE) and the molten metal filling procedure can be referred to [28].

**Figure 4:**
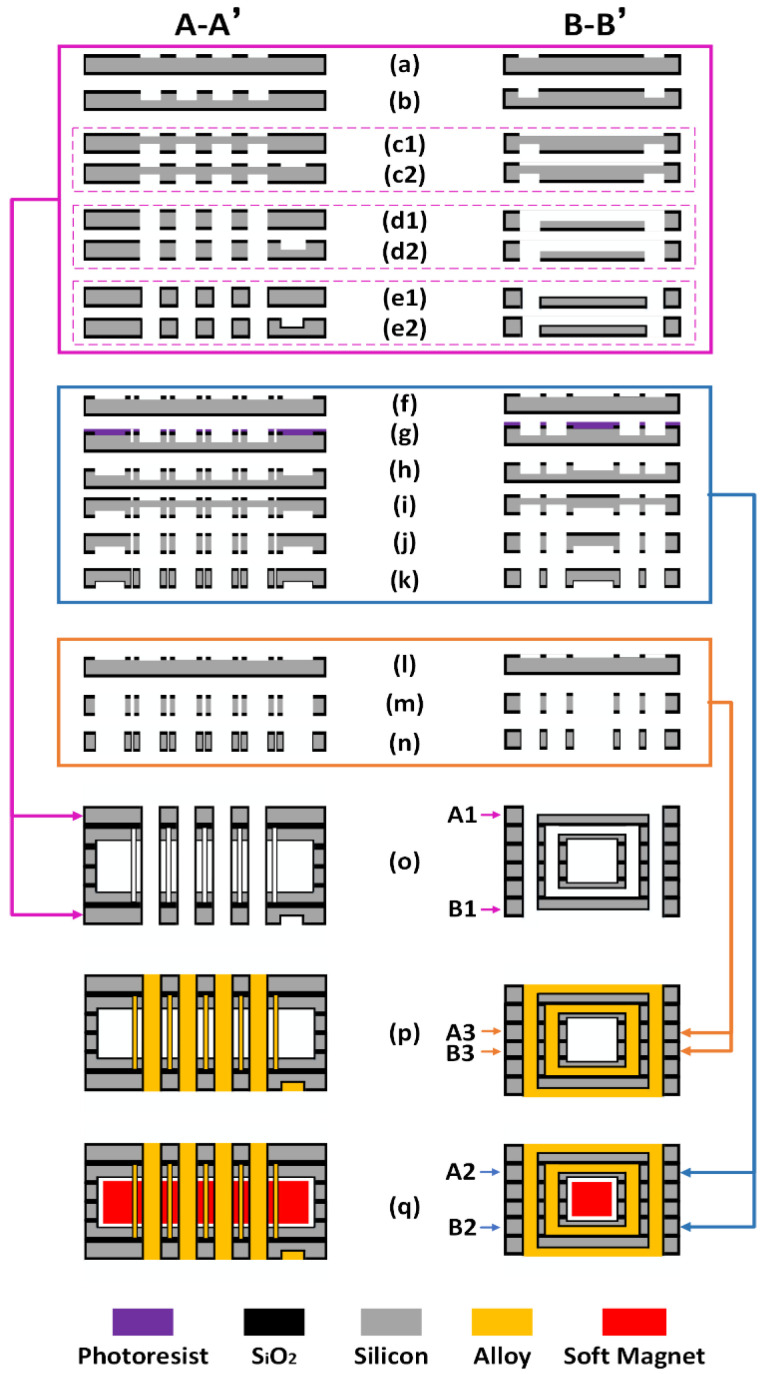
Fabrication process of the silicon-chip-based solenoid. The A-A’ and B-B’ cross sections are defined in Figure 1c. (**a**–**e2**) Formation of the grooves and vias of the outer-layer coil (for the silicon mold layers of A1 and B1); (**f**,**g**) Formation of the vias of the inner-layer coil (for A2 and B2); (**h**) Etching to form the channel for the magnet core; (**i**–**j**) Etching to form the grooves for the inner-layer coil; (**k**) Oxidation to insulate the wafer surface; (**l**–**n**) Etching and oxidation for the middle support layers (for A3 and B3); (**o**) Stacking the six silicon layers to form the whole mold for metal casting; (**p**) Formation of the solenoid by ZnAl-alloy casting and cooling; (**q**) Inserting two E-shaped soft magnet cores.

To form the on-chip integrated solenoidal transformers, we use ZnAl alloy as filling metal, with the melting point as 380 °C. The alloy features about 3.6 times resistivity of copper. The fabrication steps for the solenoidal transformers are shown in Figure 4. Please refer to Figure 4o–q, the silicon mold layers are numbered from top to bottom as A1, A2, A3, B3, B2 and B1. The entire fabrication processes shown in Figure 4 are achieved by MEMS fabrication technology and the new micro-casting technique. The steps from (a) to (n) in Figure 4 are MEMS fabrication processes. Among them, the steps from (a) to (e2) are for the silicon layers of A1 and B1, from (f) to (k) are for A2 and B2, from (l) to (n) are for A3 and B3. The steps from (o) to (p) are micro-casting fabrication processes. The A-A’ and B-B’ cross sections in Figure 4 can be referred to the indication in Figure 1c. The fabrication details are described as follows:

(a) 2 μm-thick SiO2 is thermally grown and patterned to form etching windows for the vias of the outer-layer top-coil in A1 silicon wafer and the outer-layer bottom-coil in B1 silicon wafer.

(b) The vias are etched by using DRIE technique.

(c1) The 2 μm-thick SiO2 at wafer backside is patterned to form the etching windows for the grooves in A1.

(c2) The SiO2 at the wafer backside is patterned to form the etching windows for the grooves and electrode of B1.

(d1) The grooves in A1 are etched by using DRIE. Meanwhile, the through-silicon vias in A1 are also etched through.

(d2) The grooves and electrode in B1 are etched by using DRIE. At the same time, the through-silicon vias of B1 are etched through.

(e1)–(e2) A1 and B1 are oxidized again to secure electric isolation between the metal wires and the silicon substrate for the outer-layer coils. The SiO_2_ thickness is 1 μm.

(f) The SiO_2_ is patterned to form the etching windows for the vias of the primary top-coil in A2 and the primary bottom-coil in B2.

(g) With photoresist as mask, the vias of A2 and B2 are etched by using DRIE technique.

(h) The residual photoresist is moved away. Then the cavity for the magnet accommodating channel and the vias of A2 and B2 are DRIE etched simultaneously.

(i) The SiO_2_ layers on the backsides of A2 and B2 are patterned and etched to form the windows for the following groove etching.

(j) The grooves of A2 and B2 are etched by using DRIE. The through-silicon vias are also etched through.

(k) A2 and B2 are oxidized again to secure isolation between the metal wires and the silicon molds for inner-layer coils. The grown SiO_2_ thickness is 1 μm.

(l) The SiO_2_ is patterned to form the etch windows for the vias of the middle support layers of A3 and B3.

(m) Through-silicon vias are etched through with DRIE process.

(n) A3 and B3 are oxidized again to secure the electrical insulation between the metal wires and the middle-support silicon molds.

(o) The six silicon molding wafers are aligned and stacked together to form an assembled silicon mold, and the channel for the soft magnet core is located at the center of the assembled silicon mold.

(p) The formed grooves and the vias in the stacked silicon mold are filled with the molten ZnAl alloy. After cooling, the on-chip integrated 3D solenoids are formed.

(q) The two E-shaped soft magnetic cores are inserted into the channel.

**Figure 5:**
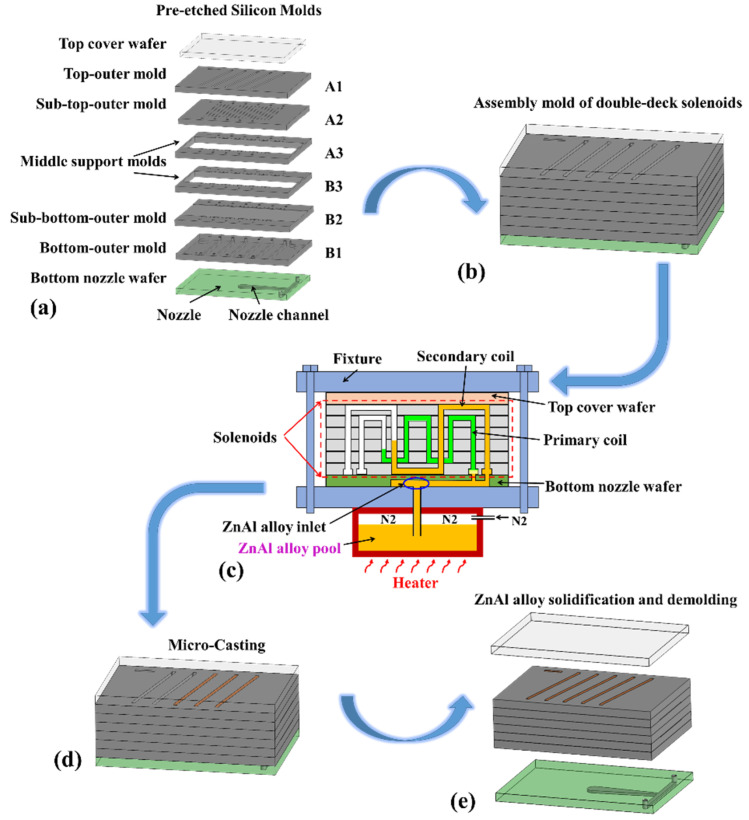
Schematic of the molten metal casting processes, including molds assembling in (**a**,**b**), alloy filling (**c**,**d**), solidification and wafer demolding (**e**).

At the casting step (p) where the ZnAl alloy is filled, the technical details can be referred to our previously published work in [28]. For better understanding, a schematic of the metal casting process is shown in Figure 5, where both the inner-deck and outer-deck solenoid coils can be fully filled with ZnAl alloy. The pre-etched silicon molds are first aligned and stacked together to form one assembly mold, and then the assembly molten filling mold is put into the micro-casting equipment for alloy filling (shown in Figure 5a–c). The alloy features a melting point as low as 380 ℃. Pressured by nitrogen gas, the molten ZnAl alloy flows out of the alloy pool and is injected into the solenoid mold through the nozzles. Within 6 min, the solenoid mold can be fully filled with the ZnAl alloy. After cooling to room temperature, the wafer containing many formed solenoids can be demolded from the top cover wafer and the bottom nozzle wafer. The casting process can be repeatedly implemented for the next silicon mold wafer (shown in Figure 5e).

**Figure 6:**
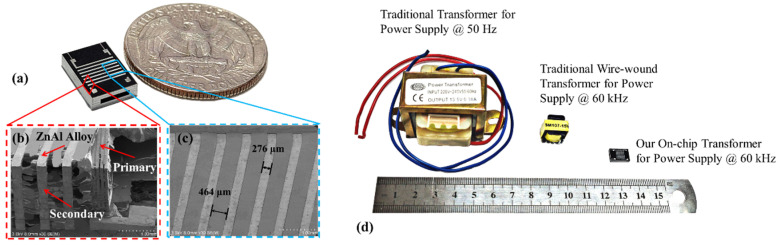
Fabricated 3D metal solenoid in a silicon chip. (**a**) Photograph showing the transformer chip. (**b**) Close-up view of the solenoids, where the primary solenoid is surrounded by the secondary solenoid; (**c**) Enlarged top view showing the ZnAl-alloy wires; (**d**) Comparison between the traditional wire-wound transformers and our on-chip transformer.

The fabricated 3D solenoid for on-chip power supply transformer is shown in Figure 6. Figure 6a is a photograph of the solenoid-contained silicon chip that is saw-diced from a 4-inch wafer, where the seven turns of secondary solenoid coil can be seen. The device occupies a space of  8.5 mm×6.6 mm×2.5 mm. The turn number of the primary solenoid is 45. Figure 6b shows the close-up view of the solenoid, where both the partially primary metal and the secondary metal can be seen. The total six silicon layers are tightly fixed by the 3D metal solenoid. The inner solenoid (primary coils) wounds around four layers of silicon substrate, and the outer solenoid interleaves six layers of silicon. The cross-sectional dimensions of the channel for accommodating the soft magnet core are  5.0 mm×0.9 mm. As shown in Figure 5c, the metal width and the space of the secondary solenoid wires are 276 μm and  464 μm, respectively. The volume is greatly reduced to 140 ${\mathrm{mm}}^{3}$ (689 ${\mathrm{mm}}^{3}$ with the soft magnetic core included) while the volume of normal commercial wire-wound 60 kHz transformer or 50 Hz transformer is 2856${\;\mathrm{mm}}^{3}$ or 55,080${\;\mathrm{mm}}^{3}$, respectively. The internal resistance of the primary solenoid is tested as 1.82 Ω and that of the secondary solenoid is 0.16 Ω. The inductance of the primary solenoid with the air core is tested as 2.9 μH and that of the secondary solenoid is 0.1 μH. With the permalloy soft magnetic core inserting into the central channel of the transformer, the inductance of the primary solenoid is tested as 37.4 μH and that of the secondary solenoid is 1.1 μH.

## 4. Testing Results

**Figure 7:**
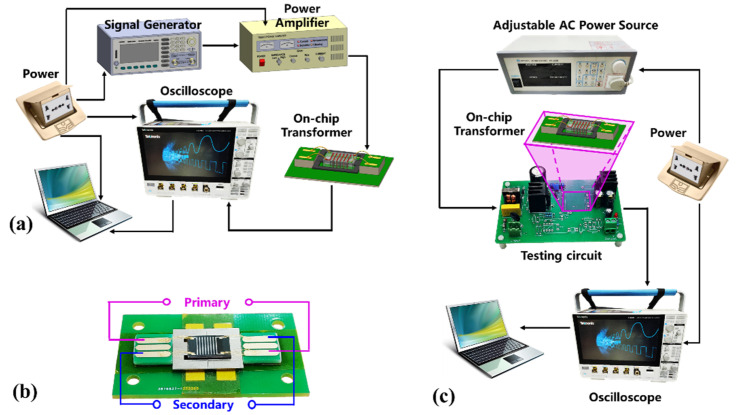
Schematic of the two testing systems. (**a**) Testing apparatus for verifying the transforming function by turns ratio; (**b**) Fabricated transformer prototype for testing; (**c**) Testing setup to confirm the transformer performance for application of power supply.

Two kinds of testing platforms are designed to assess the performance of the on-chip transformer prototype. The first one is schematically shown in Figure 7a, where continuous alternative voltage signal with frequency of 60 kHz is generated by a waveform generator (Agilent Technologies, Santa Clara, CA, USA) and then amplified by a power amplifier (TIRA, Power Amplifier BAA 120). The amplified alternative voltage signal is applied to the primary solenoid of the on-chip transformer (magnified in Figure 7b) to verify the voltage transforming performance. An oscilloscope (Tektronix, 3 series mixed domain oscilloscope) is used to record and store the testing data of the transformer.

In order to further test the performance for power supply applications, the chip-style transformer prototype is connected to a testing circuit (shown in Figure 7c). The AC voltage with an adjustable voltage amplitude at 50 Hz/60 Hz is generated by the adjustable AC power source (IVYTECH, AC power source, APS–4000B). Next, the AC voltage is input to the testing circuit. The output voltage waveforms are captured and displayed by the oscilloscope.

**Figure 8:**
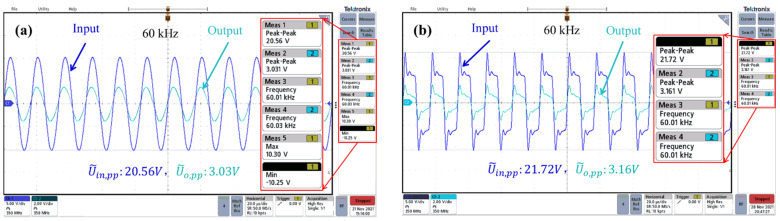
Dynamic response of the transformer under AC input at 60 kHz. (**a**) Sinusoidal input voltage signal and the peak-to-peak output voltage. (**b**) Rectangular input voltage signal and the peak-to-peak output voltage.

By using the testing setup shown in Figure 7a, the voltage transforming performance of the silicon-chip transformer is obtained when sinusoidal or rectangular voltage signals at 60 kHz are applied to the primary coil, respectively. Shown in Figure 8a, when the peak-to-peak value of sinusoidal voltage is 20.56 V, the induced peak-to-peak value voltage of the secondary solenoid is 3.03 V. When the input peak-to-peak rectangular voltage is 21.72 V, the induced peak-to-peak voltage of the secondary solenoid is 3.16 V (shown in Figure 8b). The testing ${\tilde{U}}_{in}/{\tilde{U}}_{o}$ value of about 6.8 generally agrees with the designed turn ratio of 6.4, with the slightly higher ratio possibly due to the loss of the transformer.

**Figure 9:**
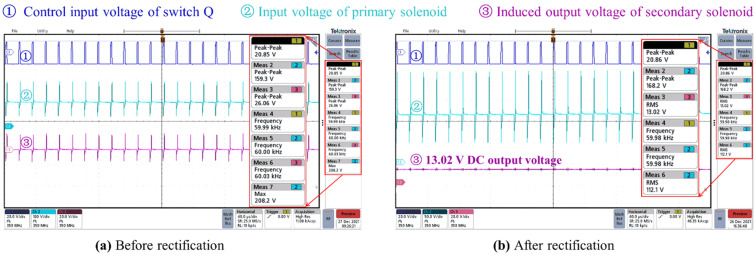
Testing results by connecting the on-chip transformer to the testing PCB circuit when input AC voltage is 110 V at 50 Hz/60 Hz. (**a**) The induced AC output voltage of the secondary solenoid before rectification; (**b**) DC output voltage after rectification.

**Figure 10:**
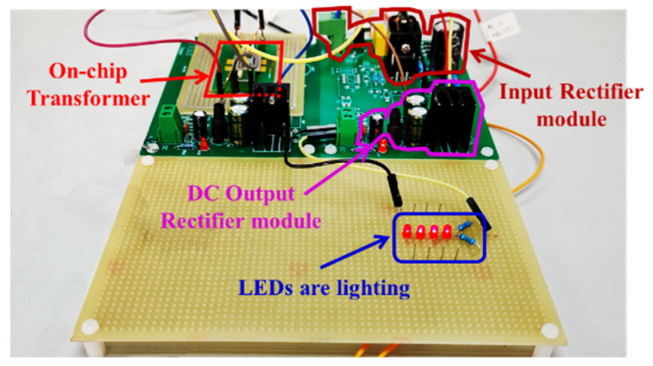
Preliminarily testing the power supply function of the transformer by directly lighting four LEDs connected in series.

To further evaluate the power supply performance, the transformer is connected to the testing circuit shown in Figure 7c. The voltage (marked as curve ① in Figure 9) is the control voltage waveform with the duty cycle of 10%. When the RMS value of the AC input voltage (marked as curve ② in Figure 9) is 110 V at 50 Hz/60 Hz, the induced AC output voltage of the secondary solenoid is shown as curve ③ in Figure 9a. After rectification, the DC output voltage of 13.02 V is obtained and shown with the curve ③ in Figure 9b. To verify the power supply function of the transformer, we connect LEDs at the output terminal of the transformer. As shown in Figure 10, the four LEDs connected in series are lit, i.e., well-powered by the DC output of the on-chip transformer.

The measured inductance of the proposed chip-style transformer has been compared with that of previously reported transformers or inductors in Table 3. It is obvious that the inductance density of solenoid is even 2714 times higher than that of the published devices, thanks to the newly micro-casting fabrication technique to fabricating the chip-style 3D solenoids for the proposed transformer.

## 5. Conclusions

A chip-style 3D metal solenoidal transformer is proposed and developed to achieve AC-DC conversion for integrated power supply applications in this article. With the new fabrication technology of micro-casting, the size of the chip-style transformer is as small as 8.5 mm × 6.6 mm × 2.5 mm. By connecting the chip-style transformer to the testing circuit of switch mode power supply operating at 60 kHz, the DC voltage of 13.02 V is obtained when the input is 110 V at 50 Hz/60 Hz. Furthermore, the on-chip 3D solenoidal transformer can light four LEDs in the power supply testing. The proposed transformer for power supply applications has been integrated on the chip by batch-fabricated, which is the most difficult electrical component to integrate on the chip in current power supplies. If the other semiconductor devices/components for electric power circuit could be further integrated in the silicon chip that already contains the transformer, a chip-style integrated power-supply module would be realized in the future.

## Figures and Tables

**Table 1:** Specification of symbols listed in Figure 2.

Symbol	Value
${\tilde{U}}_{in}$	The input AC voltage at 50 Hz or 60 Hz
${\bar{U}}_{i}$	DC input voltage after ${\tilde{U}}_{in}$ is rectified by Rectifier bridge $B_{1}$
${\bar{U}}_{o}$	DC output voltage after rectification
$I_{p}$	Current flowing through the primary solenoid
$I_{s}$	Current flowing through the secondary solenoid
$B_{1}$	Rectifier bridge for the input AC voltage ${\tilde{U}}_{in}$
$D_{1}$	Diode 1 for rectifier bridge $B_{1}$
$D_{2}$	Diode 2 for rectifier bridge $B_{1}$
$D_{3}$	Diode 3 for rectifier bridge $B_{1}$
$D_{4}$	Diode 4 for rectifier bridge $B_{1}$
$D_{5}$	Diode 5 for clamp circuit
$D_{6}$	Diode 6 for DC output rectification
$C_{1}$	Capacitor 1 for input voltage filtering
$C_{2}$	Capacitor 2 for clamp circuit
$C_{3}$	Capacitor 3 for output voltage filtering
R	Resistance for clamp circuit
$N_{p}$	Primary solenoid of the transformer
$N_{s}$	Secondary solenoid of the transformer
Q	Switch transistor

**Table 2:** Parameters of the transformer.

Parameter	Symbol	Value
Turns of primary solenoid coils	$N_{p}$	45
Width of primary coil	$w_{p}\;\left(\mu m\right)$	86
Depth of primary coil	$t_{p}\;\left(\mu m\right)$	290
Turns of secondary solenoid coils	$N_{s}$	7
Width of secondary coil	$w_{s}\;\left(\mu m\right)$	276
Depth of secondary coil	$t_{s}\;\left(\mu m\right)$	290
Inductance of the primary solenoid with air core	$L_{p,air}\left(\mu H\right)$	3.01
Inductance of the secondary solenoid with air core	$L_{s,air}\left(\mu H\right)$	0.13
Width of the channel for magnetic core	$w_{c}\left(\mathrm{mm}\right)$	5
Height of the channel for magnetic core	$h_{c}\left(\mathrm{mm}\right)$	0.92
Length of the transformer chip	$L_{T}\;\left(\mathrm{mm}\right)$	8.5
Width of the transformer chip	$W_{T}\;\left(\mathrm{mm}\right)$	6.6
Thickness of the transformer chip	$T_{T}\;\left(\mathrm{mm}\right)$	2.5
Switching frequency of switch Q	$f_{s}\left(\mathrm{kHz}\right)$	60
Input AC voltage	${\tilde{U}}_{in}\left(V\right)$	110
Frequency of input AC voltage	$f_{in}\left(\mathrm{Hz}\right)$	50 or 60

**Table 3:** Comparison with some recently published transformers/inductors.

**Reference**	**Structure Type of Transformer Coils**	**In-Substrate or** **On-Substrate**	**Number of Turns** **(*N_p_*:*N_s_*)**	**Working Frequency**	**Volume** **(cm^3^)**	**Inductance Density of Primary Coils** **(** **μ** **H/cm^3^)**	**Inductance Density of Secondary Coils** **(** **μ** **H/cm^3^)**
This work	Solenoid	In-substrate	45:7	60 kHz	0.689	54.3	1.6
[29]	3D-printedtoroid	On-substrate	19:12	40.68 MHz	14.2	0.02	0.007
[15]	Toroid	Concave-suspending in substrate	25	30–72 MHz	0.3 mm^3^	13.6–17.3	-
[30]	Solenoid	In-substrate	20	21 MHz	4 mm^3^	18.3–21.7	-
[28]	Solenoid	In-substrate	20	>1 MHz	1.496 mm^3^	10.2	-
[31]	Toroid	Concave-suspending in substrate	25	40–10 MHz	29.7 mm^3^	1.16–2.02	-
